# Prognostic impact of sarcopenia in patients with head and neck cancer treated with surgery or radiation: A meta-analysis

**DOI:** 10.1371/journal.pone.0259288

**Published:** 2021-10-29

**Authors:** Yukinori Takenaka, Norihiko Takemoto, Ryohei Oya, Hidenori Inohara

**Affiliations:** Department of Otorhinolaryngology-Head and Neck Surgery, Osaka University Graduate School of Medicine, Osaka, Japan; University of California, Davis, UNITED STATES

## Abstract

**Background:**

The objective of this study was to compare the prognostic impact of sarcopenia in patients with head and neck cancer (HNC) treated with surgery or radiation.

**Methods:**

We systematically searched electronic databases to identify articles reporting the impact of sarcopenia on the prognosis of patients with HNC. Hazard ratios (HRs) and 95% confidence intervals (CIs) for overall survival (OS), disease-free survival (DFS), and disease-specific survival (DSS) were extracted and pooled. HR according to treatment modality were estimated using random-effects models. Statistical analyses were carried out using the Comprehensive Meta-Analysis software.

**Results:**

In total, 18 studies enrolling 3,233 patients were included. Sarcopenia was associated with poor OS in both surgery and radiotherapy groups (hazard ratio [HR] 2.50, 95% confidence interval [CI] 1.95–3.21; HR 1.63, 95% CI 1.40–1.90, respectively). The HR was significantly higher in the surgery group than in the radiotherapy group (*p* = 0.004), with similar results obtained for DFS (HR 2.59, 95% CI 1.56–4.31; HR 1.56, 95% CI 1.24–1.97 for the surgery and radiotherapy groups, respectively) and DSS (HR 2.96, 95% CI 0.73–11.95; HR 2.67, 95% CI 1.51–4.73 for the surgery and RT groups, respectively).

**Conclusions:**

Sarcopenia was a poor prognostic factor for HNC, regardless of the treatment modality. However, the adverse effects of sarcopenia on survival were more prominent in the surgery group than in the radiotherapy group. Sarcopenia assessment is required for appropriate treatment decision-making.

## Introduction

Head and neck cancer (HNC) is the sixth most common cancer worldwide [1]. Either surgery or radiotherapy is used for treatment of early-stage HNC, whereas surgery or chemoradiation therapy (CRT) is used for the treatment of locoregionally advanced HNC [2]. Surgery for advanced HNC often destroys anatomical structures and can result in permanent functional impairments. On the other hand, radiotherapy can preserve organs and their functioning. However, late adverse effects of chemoradiation therapy can cause failure of functional organ preservation [3]. Furthermore, CRT requires a long treatment period, and as a result, some patients cannot complete the planned treatment regimen. To determine the optimal treatment strategy for these patients, an assessment of the factors associated with treatment efficacy is warranted. Tumor characteristics, including histologic type, extent of spread, volume, and human papillomavirus (HPV) status, should be considered. In addition, patient characteristics, such as age, comorbidities, and nutritional status, should be taken into account.

Nutritional parameters, including hematologic markers like hemoglobin concentration, lymphocyte count, and serum albumin concentration, as well as the body mass index (BMI), have been investigated for their associations with HNC prognosis [4]. A previous study has indicated that the impact of BMI on HNC prognosis might depend on the type of cancer treatment [5]. However, BMI cannot be employed for treatment decisions, partly because of the obesity paradox [6]. In addition, BMI cannot discriminate between different body compositions, such as muscle or fat mass. Thus, body composition assessments using dual-energy X-ray absorptiometry, bioelectrical impedance analysis, and computed tomography (CT) have gained attention.

Sarcopenia is a skeletal muscle disorder characterized by low muscle strength, quality, and quantity [7, 8]. Patients with head and neck cancer are prone to sarcopenia because of swallowing disability caused by the primary tumor, comorbidities resulting from habitual drinking and smoking, old age, and cancer-induced catabolism [9]. The prevalence of sarcopenia in patients with HNC ranges from 6.6% to 70.9%, depending on the patient population, diagnostic procedures for sarcopenia, and cut-off values [9]. A recent meta-analysis revealed sarcopenia as an independent prognostic factor for overall survival (OS) in patients with HNC treated with radiotherapy (RT) [10]. In contrast, findings on the prognostic impact of sarcopenia in patients with HNC treated surgically have varied largely among studies, with no meta-analysis studies on the topic.

In this study, we aimed to investigate the prognostic impact of sarcopenia in patients with HNC and to compare its prognostic ability in HNC patients treated with surgery versus those treated with radiotherapy.

## Materials and methods

### Search strategy

This study was conducted in accordance with the guidelines for the Preferred Reporting Items for Systematic Reviews and Meta-Analyses [11]. We searched for published studies related to the association between sarcopenia and HNC in the following electronic databases: PubMed www.ncbi.nlm.nih.gov/pubmed‎, Scopus www.elsevier.com/online-tools/scopus, and Ichushi*-*Web https://search.jamas.or.jp which contains bibliographic information and abstracts of articles in Japanese journals, from database inception to February 7, 2021. The search terms were #1: "head and neck" or "larynx" or "laryngeal" or "oropharynx" or "oropharyngeal" or "hypopharynx" or "hypopharyngeal" or "oral" or "tongue" or "parotid" or "salivary gland" or "nasal" or "paranasal"; #2: "tumor" or "malignancy" or "cancer"; and #3: “sarcopenia” or “sarcopenic” or “muscle index” or “muscle mass” or “muscle depletion” or “muscular atrophy” or “muscle strength” or “muscle quality” or “muscle quantity” or “myosteatosis” or “myopenia.” The detailed search terms are provided in S1 File. References in the retrieved articles were manually searched for associated studies. The protocol for this meta-analysis is available in the UMIN (registration code: UMIN000043139).

### Study selection

The inclusion criteria for this study were as follows: (1) studies reporting the prognostic impact of sarcopenia in HNC; (2) sarcopenia defined using muscle mass on computed tomography (CT) imaging; (3) hazard ratios (HR) and 95% confidence intervals (CI) according to surgery or RT for disease-free survival (DFS), disease-specific survival (DSS), and/or overall survival (OS) were shown or estimated from the published data; and (4) the histological type of tumors was considered to be predominantly squamous cell carcinoma (SCC). The exclusion criteria were as follows: (1) non-human studies, case reports, or reviews; (2) studies in languages other than English or Japanese; (3) muscle mass or radiodensity not dichotomized to define sarcopenia, and (4) studies on thyroid cancer, nasopharyngeal cancer, or salivary gland cancer in which SCC and non-SCC were analyzed together. Two of the authors (YT and RO) independently evaluated the electronically searched titles. All potentially relevant publications were retrieved.

### Data extraction

The following data were extracted: first author’s name; year of publication; institution and country; number of patients; primary tumor sites; disease stage; treatment modality; patient age; HPV status; diagnostic measures for sarcopenia; cut-off methods; cut-off values, 95% CIs, and *p*-values for OS and DSS. The HRs, 95% CIs, and *p*-values were preferentially extracted from multivariate analyses; if unavailable, HRs were extracted from univariate analyses. The Quality in Prognostic Studies (QUIPS) tool [12] was used to assess the risk of bias in included studies.

### Statistical analysis

Meta-analyses were conducted using Comprehensive Meta-Analysis Version 2 (Biostat, Englewood, NJ, USA). Because of heterogeneity between studies, a random-effects model using the DerSimonian and Laird method was implemented [13, 14]. A comparison of the HRs between the surgery and RT groups was performed for DFS, DSS, and OS. If a study had investigated patients who had undergone surgery followed by (chemo-) radiation therapy (C)RT, the patients were grouped into the surgery group. In addition, subgroup analyses were conducted based on study region, sarcopenia assessment methods, and analysis type. In the subgroup analysis for region, studies were divided into those from Western or Eastern countries. For the sarcopenia assessment method subgroup analysis, studies were divided into two groups: those using the skeletal muscle mass index (SMI) calculated from the muscle area at the third lumbar (SMI- L3) spine level versus those using the SMI at the third cervical (SMI-C3) spine level. For the subgroup analysis according to analysis type, combined HRs for adjusted HRs and unadjusted HRs were estimated separately. Publication bias was assessed using a funnel plot and tested using Egger’s regression intercept test. Heterogeneity was assessed using the Cochran Q test and *I*^2^ statistics. All statistical tests were two-sided, and a *p*-value of <0.05 was considered statistically significant.

## Results

### Literature search results and study characteristics

**Fig 1:**
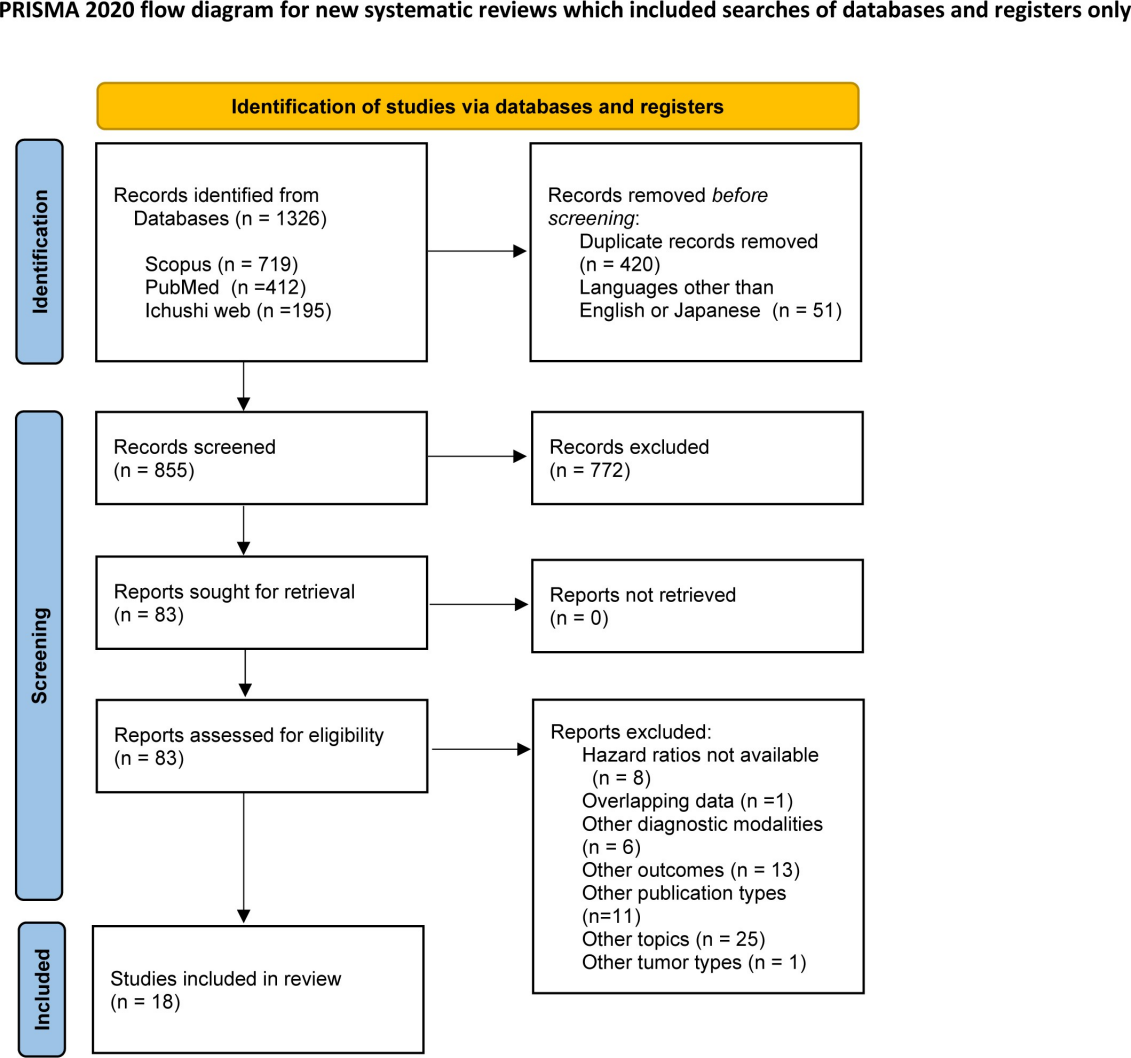
Flow diagram of article selection.

The electronic database searches retrieved 1,326 records (Fig 1). We screened the titles and abstracts of these studies and identified 83 potentially eligible studies. The full-text versions of these 83 studies were then inspected according to the exclusion criteria, and 18 studies involving 3,233 patients were determined to be eligible for inclusion in this study [15–32] (Table 1). In these 18 studies, seven investigated patients treated with surgery [17, 20, 25–27, 31, 32] and 10 investigated patients treated with RT [15, 16, 18, 19, 21–24, 29, 30]. One study investigated both surgically and radiotherapeutically treated patients [28]. The study by Yoshimura et al. [31] used the psoas muscle mass index, and the study by Choi et al. [21] used the skeletal muscle area of the neck. The remaining 16 studies used skeletal muscle mass index (SMI), with seven [15, 18, 23, 25, 27–29] and nine [16, 17, 19, 20, 22, 24, 26, 30, 32] studies measuring the muscle area at the L3 and C3 spine levels, respectively. Eight studies [16, 18, 19, 22–24, 29, 30] in the RT group and six studies [17, 20, 25–27, 31] in the surgery group reported stage distribution between stage I, II and stage III, IV disease. Among them, 79.0% of the 1772 patients in the RT group had stage III, IV disease, whereas 63.4% of the 1026 patients in the surgery group had stage III, IV disease.

**Table 1:** Study characteristics.

Year	Author	Country	No of patients	Site	Stage III/IV (%)	Age median [range} or mean ± SD	Treatment	Outcomes	Sarcopenia			
									Assessment of muscle mass	Spine level	Cutoff value (cm^2^/m^2^)	Prevalence (%)
2016	Grossberg	USA	190	HP, L, OC, OP, other	NA	57.7 ± 9.4	(C)RT	DSS, OS	SMI	L3	52.4 for male 38.5 for female	35.3
2017	Wendrich	Netherlands	112	HP, L, OP, other	100.0	54.5 ± 9.4	Platinum-based CRT	OS	SMI	C3	43.2	54.5
2018	Bril	Netherlands	245	HP, L	67.7	64.7 ± 9.1	Total laryngectomy	OS	SMI	C3	43.2	44.5
2018	Cho	Korea	221	HP, L, OC, OP, other	100.0	59 [18–94]	CRT	OS	SMI	L3	49 for male 31 for female	48.0
2019	Ganju	USA	246	HP, L, OP, other	100.0	60 [19–88]	(C)RT	OS	SMI	C3	43 for male, BMI<25 53 for male, BMI>25 41 for female	58.1
2020	Ansari	Netherlands	78	OC	94.9	62.4 ± 10.2	Surgery with free fibula flap reconstruction	OS	SMI	C3	43.2	61.5
2020	Choi	Korea	79	HP, OC, OP, other	NA	58.5 ± 12.8	(C)RT	DFS, OS	Skeletal muscle area from the level of C3 to the level of the first rib 607cm^3^ for male 450cm^3^ for female			13.9
2020	Endo	Japan	159	L, HP, OP	86.8	65 [43–85]	CDDP-based CRT	OS	SMI	C3	12.3	NA
2020	Findlay	Australia	79	HP, L, OC, OP, other	79.7	61.0 ± 11.6	(C)RT	OS	SMI	L3	43 for male, BMI<25 53 for male, BMI>25 41 for female	53.2
2020	Huiskamp	Netherlands	91	HP, L, OP, other	95.6	62.18 ± 7.22 for sarcopenia 63.33 ± 7.78 for non-sarcopenia	Cetuximab + RT	DFS, OS	SMI	C3	45.2	74.7
2020	Jung	Korea	190	L, HP, OC, OP	68.4	71.9 ± 5.1	Surgery	DFS, OS	SMI	L3	52.4 for male 38.5 for female	33.7
2020	Lee	Taiwan	174	OC	100.0	51 [45–59]	Surgery followed by CRT	DFS, OS	SMI	C3	52.4 for male 36.2 for female	31.0
2020	Makiguchi	Japan	111	OC	70.3	60 [23–76]	Surgery with free flap reconstruction	DFS, OS	SMI	L3	36.02 for male 31.76 for female	41.4
2020	Olson	USA	245	OP	T1-2 N0-2	62.3 ± 7.8	RT, surgery	DSS, OS	SMI	L3	52.4 for male 38.5 for female	55.1
			142			62.1 ± 7.5	Surgery					50.0
			103			62.5 ± 8.2	(C)RT					62.1
2020	Shodo	Japan	41	HP, L, OP, other	85.4	62.4 ± 8.3	CDDP-based CRT	DSS, OS	SMI	L3	39.7	26.8
2020	van Rijin-Dekker	Netherlands	744	HP, L, OC, OP, other	69.4	66 ± 10 for sarcopenia 62 ± 10 for non-sarcopenia	(C)RT	DFS, OS	SMI	C3	42.4 for male 30.6 for female	25.4
2020	Yoshimura	Japan	103	OC	51.5	68 [59–77]	Surgery	DSS, OS	PMI	L3	6.05 for male 5.097 for female	28.1
2021	Chang	Taiwan	125	OC	52.8	NA	Surgery	DFS, OS	SMI	C3	20.71	38.4

Abbreviations: BMI, body mass index, C3, third cervical vertebra, CDDP, cisplatinum, CRT, chemoradiation therapy, DFS, disease-free survival, DSS, disease-specific survival. L3, third lumbar vertebra, HP, hypopharynx, L, larynx, NA, not available, OC, oral cavity, OP, oropharynx, OS, overall survival, SMI, skeletal muscle index, PMI, psoas muscle index, RT, radiation therapy.

### Quality assessment

**Fig 2:**
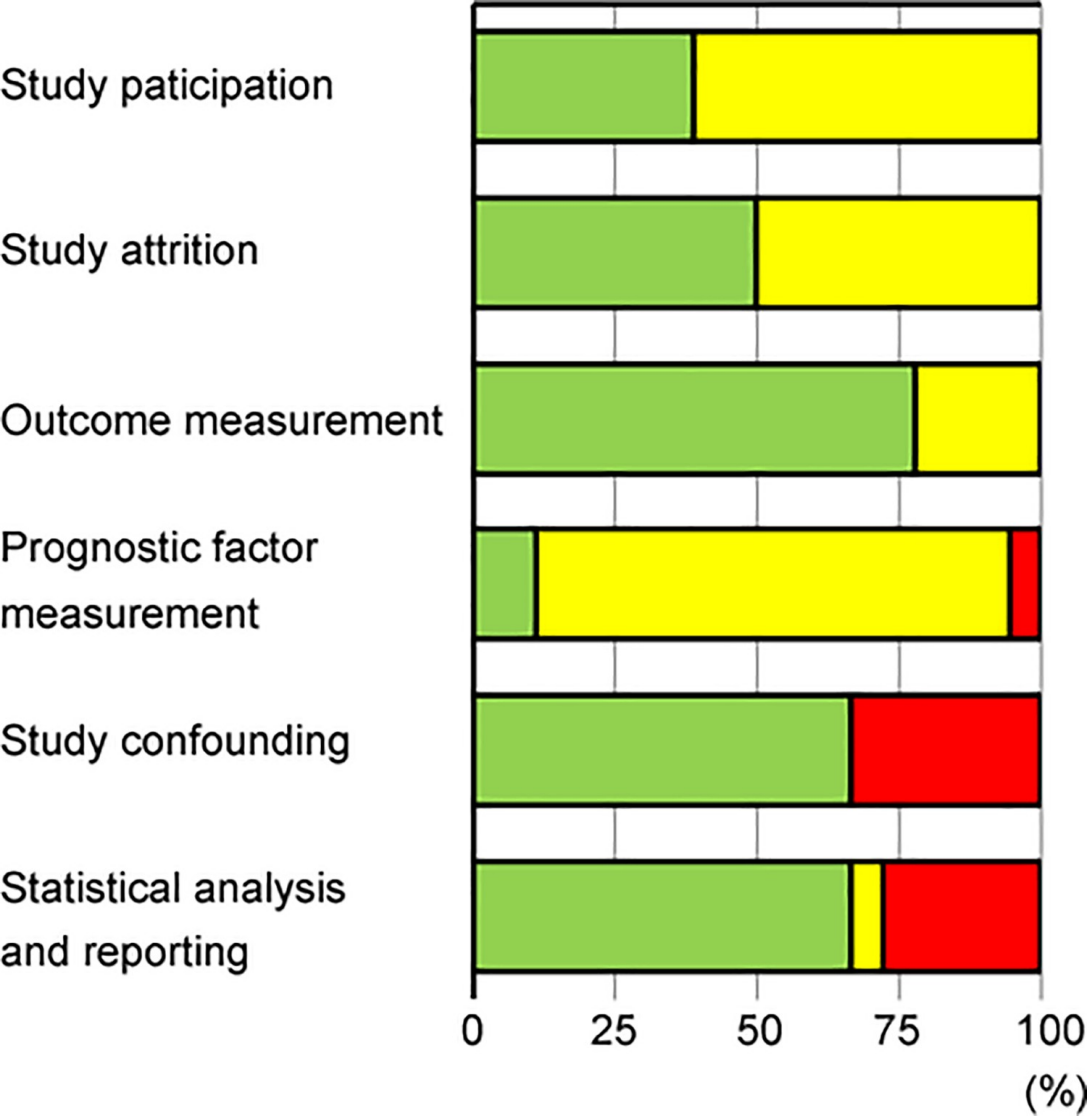
Risk-of-bias assessment for included studies. Green = low risk of bias, yellow = moderate risk of bias, red = high risk of bias.

The risk of bias in the included studies was assessed using the QUIPS tool, which included six domains: study participation, study attrition, prognostic factor measurement, outcome measurement, study confounding, and statistical analysis and reporting (S1 Table). Fig 2 summarizes the risk-of-bias assessment. Overall, the quality of the included studies was low or moderate, mainly because of their retrospective nature. In particular, the timing of sarcopenia assessment was not described or was more than 1 month before treatment in many of the studies, which resulted in a high or moderate risk of bias in the prognostic factor measurement domain. Notably, the HRs according to treatment modality were not shown in some published articles [15, 17, 18, 22, 28, 29, 31] and were therefore estimated from Kaplan-Meier curves. As a result, some well-designed studies were graded as having a high risk of bias in the study confounding and the statistical analysis and reporting domains (S1 Table).

### Sarcopenia and survival

**Fig 3:**
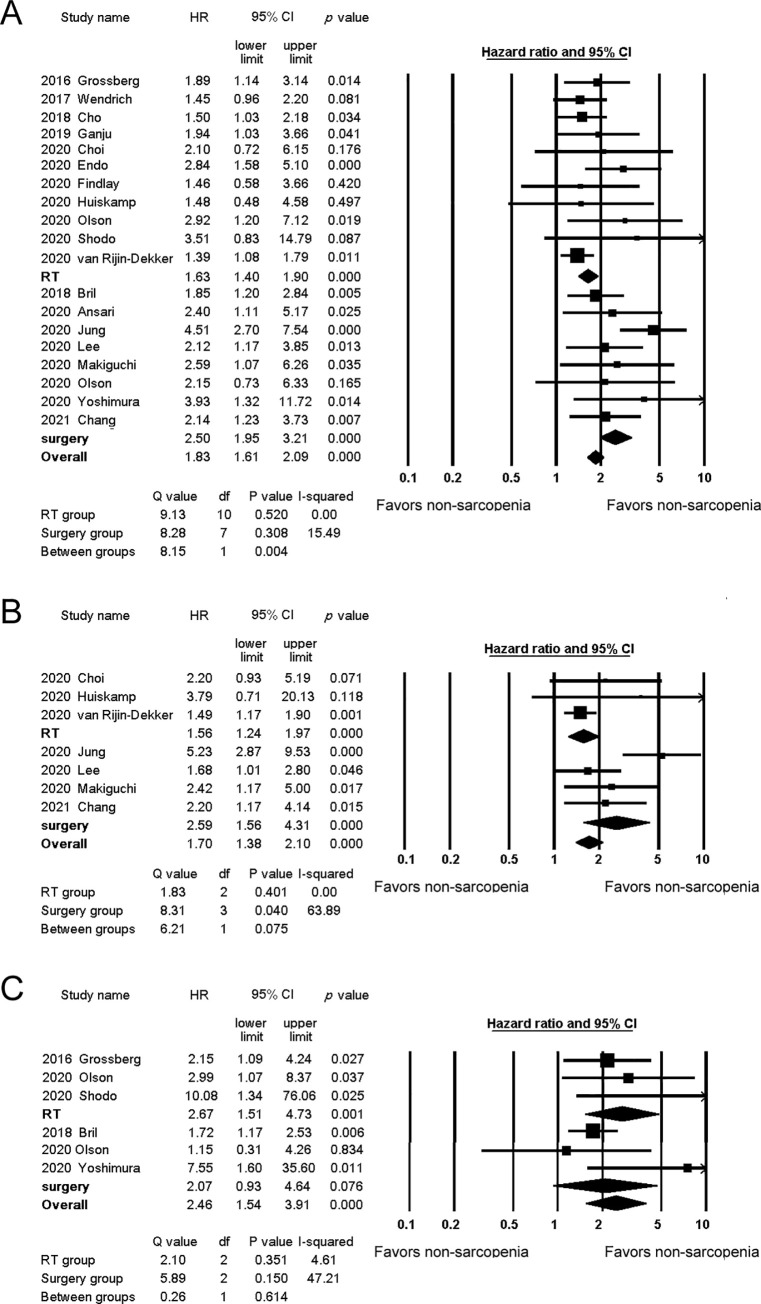
Forest plots of studies evaluating hazard ratios for sarcopenia. A. Studies evaluating sarcopenia and overall survival. B. Studies evaluating sarcopenia and disease-free survival. C. Studies evaluating sarcopenia and disease-specific survival.

The HRs for OS were reported in all of the included studies and ranged from 1.39 to 4.51. The pooled analysis results are shown in Fig 3A. The combined HR was significantly higher for the surgery group (HR 2.50, 95% confidence interval (CI) 1.95–3.21) than for the RT group (HR 1.63, 95% CI 1.40–1.90) (*p* = 0.004). To exclude the effect of confounding by stage, we conducted an analysis for only the advanced stage disease. The analysis revealed a similar result (HR 2.22, 95% CI 1.39–3.56 for the surgery group and HR 1.54, 95% CI 1.20–1.98 for the RT group).

The HRs for DFS were reported in seven studies [21, 24–27, 30, 32] and ranged from 1.49 to 5.23. The combined HRs for the surgery and RT groups were 2.59 (95% CI 1.56–4.31) and 1.56 (95% CI 1.24–1.97), respectively (Fig 3B). The difference between the two groups was not statistically significant (*p* = 0.075). The HRs for DSS were reported in five studies [15, 17, 28, 29, 31] and ranged from 1.72 to 10.08. The combined HRs were comparable between the surgery and RT groups (HR 2.96, 95% CI 0.73–11.95, HR 2.67, 95% CI 1.51–4.73, respectively) (Fig 3C), and the difference between the two groups was not statistically significant (*p* = 0.896).

### Subgroup analyses

Subgroup analyses were conducted for the study region and the sarcopenia assessment method (Table 2). When studies from Eastern and Western countries were analyzed separately, both analyses showed higher HRs for the surgery group than for the RT group. Similarly, the subgroup analysis of the sarcopenia assessment method showed higher HRs for the surgery group in both the SMI-C3 and SMI-L3 subgroups. To exclude the effect of confounding factors, we conducted an analysis for adjusted HRs. Twelve studies [15, 17, 19, 21, 23–27, 30–32] showed HRs adjusted for confounding factors, including tumor sites and stage. The combined HRs for the surgery and RT groups were 2.47 (95%CI 1.84–3.32) and 1.54 (95% CI 1.26–1.88), respectively (p = 0.009). The combined HRs for the adjusted group were lower than those for the unadjusted group.

**Table 2:** Subgroup analysis.

		No. of studies	No. of patients	HR	95% CI		Q value	I-squared	*p* value between groups
					lower limit	upper limit			
Eastern	RT	4	500	2.02	1.35	3.01	4.07	26.28	0.195
	Surgery	5	703	2.56	2.02	4.05	5.40	25.94	
Western	RT	7	1565	1.55	1.29	1.85	3.85	0.00	0.217
	Surgery	3	469	1.99	1.39	2.83	0.36	0.00	
SMI-C3	RT	5	1352	1.64	1.27	2.1	5.42	26.13	0.244
	Surgery	4	622	2.04	1.55	2.69	0.42	0.00	
SMI-L3	RT	5	634	1.77	1.34	2.28	3.05	0.00	0.007
	Surgery	3	443	3.53	2.28	5.47	2.15	6.84	
Adjusted	RT	6	1429	1.54	1.26	1.88	2.11	0.00	0.009
	Surgery	6	923	2.47	1.84	3.32	7.52	33.53	
Unadjusted	RT	5	636	1.90	1.37	2.64	6.22	35.65	0.321
	Surgery	2	245	2.90	1.34	6.24	0.59	0.00	

Abbreviations: HR, hazard ratio; CI, confidence interval; C3, third cervical vertebra, L3, third lumbar vertebra, SMI, skeletal muscle index, RT, radiation therapy.

### Publication bias

**Fig 4:**
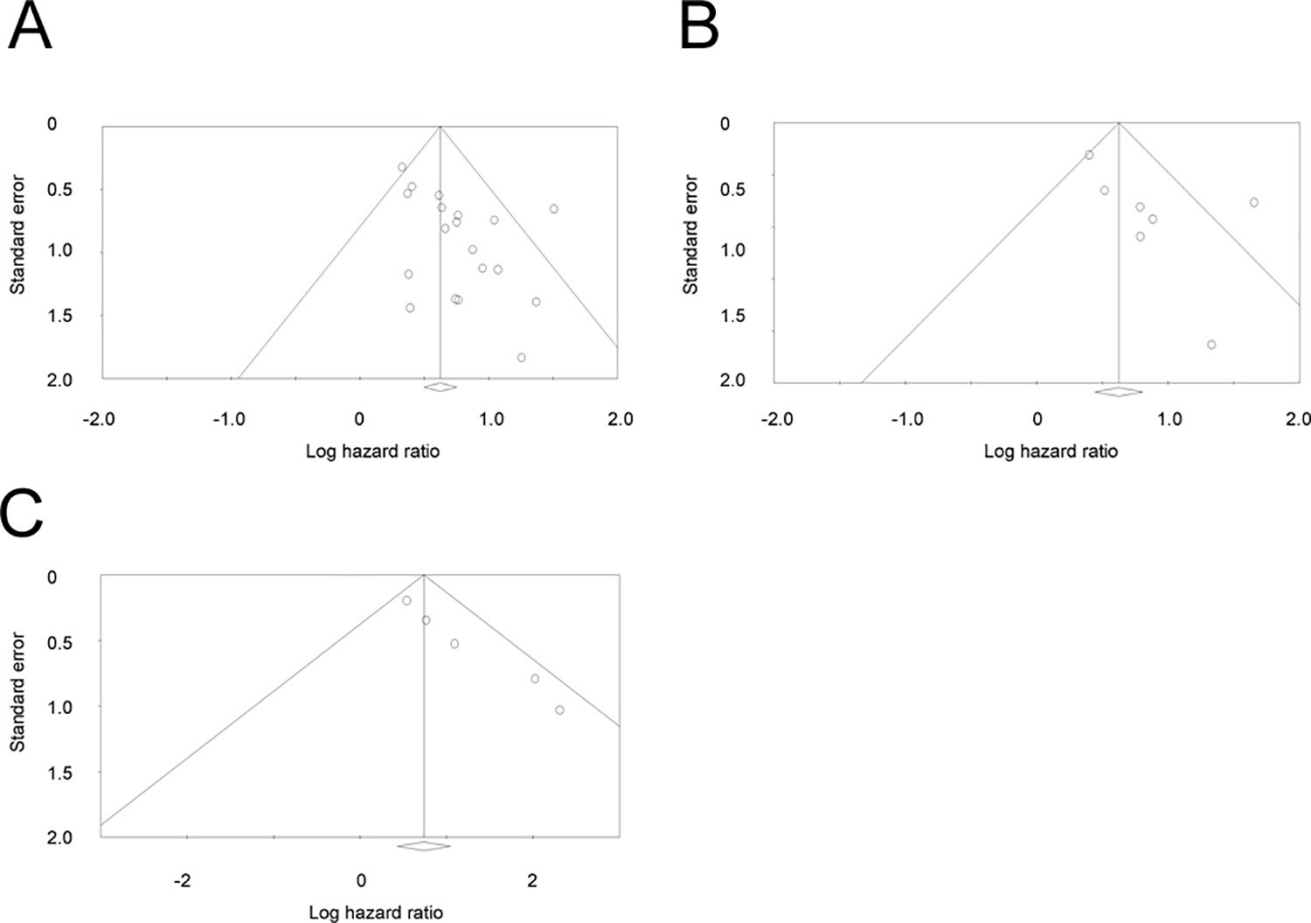
Funnel plot of hazard ratios for the relationship between sarcopenia and overall survival, disease-free survival, and disease-specific survival (A, B, and C, respectively).

Fig 4 shows a funnel plot of the HRs for OS, DFS, and DSS. Asymmetry was conspicuous in the funnel plots for DFS and DSS. *P*-values derived from Egger’s test were 0.015, 0.107, and 0.002 for OS, DFS, and DSS, respectively.

## Discussion

Sarcopenia was first described as a geriatric syndrome characterized by muscle deficiency [33]. However, patients with advanced cancer also often suffer from a loss of muscle mass and strength caused by malnutrition and altered metabolism. Thus, the term sarcopenia has been adapted to the field of oncology as well. Many studies have demonstrated an association between sarcopenia and adverse outcomes in patients with cancer. A meta-analysis by Shachar et al. showed that sarcopenia assessed using the SMI, was associated with poor OS in various types of solid cancers [34]. Three meta-analyses [10, 35, 36] have been published on the role of sarcopenia in patients with HNC. Wong et al. investigated 10 studies consisting of 2,181 patients and demonstrated that radiologically defined sarcopenia was a negative predictor of OS [35] Hua et al. investigated 11 studies involving 2,483 patients, showing poorer OS and DFS in patients with sarcopenia [36]. These two meta-analyses analyzed patients undergoing various treatment modalities. Findlay et al. analyzed data from seven studies consisting of 1,059 patients treated with RT and demonstrated that pretreatment CT-defined sarcopenia was associated with reduced OS [10]. However, there have been no related meta-analyses focusing on patients treated surgically. Several studies have reported the prognostic impact of sarcopenia on patients treated surgically [17, 20, 25–27, 31, 32]. However, these studies were retrospective cohort studies with a small sample size of patients. In addition, no study has compared the prognostic effect of sarcopenia in patients treated with surgery versus those treated with RT. In the present meta-analysis, we showed that sarcopenia is a prognostic factor for OS and DFS in patients treated with surgery and RT. However, notably, the adverse effects of sarcopenia were more pronounced in the surgery group than in the RT group.

Surgery is generally the treatment of choice for many types of resectable solid cancers. However, for HNC, two main treatment modalities exist: surgery and RT. Monotherapy with either surgery or RT is employed to treat HNC in the early stages, while surgery with adjuvant therapy or (C)RT is used for the treatment of locoregionally advanced HNC [2]. The choice between surgery or (C)RT for advanced HNC is often difficult. However, the organ-preserving approach using (C)RT has prevailed worldwide during the last few decades because it enables patients with advanced HNC to retain speech and swallowing abilities. Although functional preservation of the upper aerodigestive tract is necessary, a patient’s top priority is to be cured [37]. It remains controversial, however, whether (C)RT can achieve comparable results to surgery [38, 39]. To decide the optimal treatment strategy, clinicians should consider patient age, preference, comorbidities, tumor extent, and nodal status. In addition, our meta-analysis indicates that RT may be a better choice for treatment of sarcopenic patients with HNC.

Some potential explanations for the prognostic impact of sarcopenia are described as follows. First, sarcopenia is a surrogate for general physical status. A study on lung cancer demonstrated that sarcopenia was closely associated with performance status [40], and performance status is the most powerful prognostic factor for advanced cancer [41]. Therefore, sarcopenia, as a surrogate marker for performance status, can predict the prognosis of patients with cancer. Second, sarcopenia is associated with postoperative complications. Sarcopenic patients with laryngeal cancer have a higher incidence of pharyngocutaneous fistulas [17]. Sarcopenia was also reported to be a predictive factor for free-flap complications in patients undergoing oral cancer surgery [20]. These postoperative complications can hinder or delay adjuvant therapy, resulting in a poor prognosis. Third, a loss of muscle mass changes the characteristics of circulating myokines, which are cytokines secreted by muscle cells. Interleukin (IL)-6, a myokine, has been shown to exert an antitumorigenic effect in a mouse model [42]. Other myokines, including IL-15, IL-8, and myostatin, can also affect oncologic outcomes [43]. Fourth, sarcopenia may be associated with a progressive tumor status, with some studies showing advanced T and N classifications in sarcopenic patients [17, 27, 44]. Finally, sarcopenia increases adverse events during and after RT, including aspiration pneumonitis during CRT [22] and dose-limiting toxicity during cetuximab treatment [24]. These adverse events can lead to treatment cessation and poorer disease control [29]. In addition, sarcopenic patients experience more late toxicity events, such as xerostomia and dysphagia [30], which can affect survival and quality of life. Collectively, sarcopenia reflects the status of the patient and the tumor, and increases the risk of adverse events, all of which can lead to a poorer prognosis.

In this study, HR was higher in the surgery group than the RT group, which may be partly explained by short-term mortality. A study by Bril et al. [17] demonstrated postoperative mortality of 3.7% in patients with sarcopenia, in contrast to 0% in patients without sarcopenia. Similarly, Galli et al. [45] reported a thirty-day postoperative mortality rate of 22.2% in the sarcopenia group and 0% in the non-sarcopenia group, in which half of the deaths within 30 days were due to sepsis resulting from pharyngocutaneous fistula or aspiration pneumonitis, and the other half were caused by cardiac events. Thus, postoperative infections and complications from perioperative stress in surgically treated patients with sarcopenia result in higher mortality.

There are several methods for CT-defined sarcopenia. SMI-L3 has been the most widely used index, and its usefulness has been shown in various medical fields. However, SMI-C3 has an advantage of being assessed with routine CT scan and a disadvantage of being affected by tumor invasion into surrounding muscle [32, 44]. These two indices corelate closely, and our subgroup analysis demonstrated a similar prognostic capability between the two. Other commonly used indices for sarcopenia include PMI and skeletal muscle density (SMD). Both indices have been shown to be associated with the prognosis of several types of cancer [46]. However, only a few reports investigate their significance in HNC [23, 31]. Collectively, SMI-L3 and SMI-C3 would be recommended methods for the assessment of sarcopenia in patients with HNC.

This study had several limitations. First, the studies included in this meta-analysis were retrospective cohort studies, which may have caused considerable biases, including information and selection biases. Furthermore, the small number of patients in some studies made it impossible to conduct multivariate analyses. Second, a significant publication bias was present in the OS and DSS analyses. Although publication bias was not shown in the DFS analysis, this may have been due to the limited number of studies. Third, HPV status was considered only in the study by Olson et al [28]. HPV status is one of the most powerful prognostic factors for HNC and affects treatment decisions. Therefore, the HPV status should be included as a covariate in multivariate analyses whenever survival analyses for HNC are conducted. Fourth, we investigated the association between CT-defined muscle mass and cancer prognosis. Because of the higher adiposity of Asian people compared with Caucasians [8], muscle mass may be overestimated in studies from Asia, which might result in the difference between ethnicities. Intramuscular adiposity or myosteatosis should be assessed in future studies. Lastly, the primary tumor sites differed between the surgery and RT groups. Among the eight studies [17, 20, 25–28, 31, 32] that analyzed surgically treated patients, five [20, 26, 27, 31, 32] included oral cavity cancers only. Therefore, the different prognostic implications of sarcopenia in the surgery and RT groups might have resulted from differences in tumor sites. Also, the stage distribution differed among studies. To deal with these problems, we conducted subgroup analyses. However, individual patient data meta-analysis is required to fully address the confounders.

In the present meta-analysis, we demonstrated that sarcopenia is a prognostic factor in patients with HNC, irrespective of their treatment modality. However, the prognostic impact differed significantly between the surgery and RT groups. Thus, clinicians should incorporate sarcopenia assessments into their treatment decision-making. Future research is required to investigate whether the negative effects of sarcopenia can be negated by exercise or nutritional therapy.

## Supporting information

S1 Checklist Preferred reporting items for systematic reviews and meta-analyses 2020 checklist. (DOCX)

S1 File Search strategy. (DOCX)

S1 Table Quality in prognostic studies results for included studies. (XLSX)

S2 Table Grading of recommendations assessment, development and evaluation (GRADE) table. (XLSX)
