# Supplementary material for: Prognostic impact of sarcopenia in patients with head and neck cancer treated with surgery or radiation: A meta-analysis
Source: PLoS One. 2021 Oct 29;16(10):e0259288. doi: 10.1371/journal.pone.0259288 (PMC8555817; doi:10.1371/journal.pone.0259288)
Supplement: S1 File — (DOCX) [file pone.0259288.s002.docx]

PubMed

**"head and neck"[All Fields] OR "larynx"[All Fields] OR "laryngeal"[All Fields] OR "oropharynx"[All Fields] OR "oropharyngeal"[All Fields] OR "hypopharynx"[All Fields] OR "hypopharyngeal"[All Fields] OR "oral"[All Fields] OR "tongue"[All Fields] OR "parotid"[All Fields] OR "salivary gland"[All Fields] OR "nasal"[All Fields] OR "paranasal"[All Fields]) AND ("tumor"[All Fields] OR "malignancy"[All Fields] OR "cancer"[All Fields] OR "carcinoma"[All Fields]) AND ("sarcopenia"[MeSH Terms] OR "sarcopenia"[All Fields] OR "sarcopenic"[All Fields] OR "muscle mass"[All Fields] OR "muscle index"[All Fields] OR "muscle depletion"[All Fields] OR "muscle atrophy"[All Fields] OR "muscle strength"[All Fields] OR "muscle quality"[All Fields] OR "muscle quantity"[All Fields] OR "myosteatosis"[All Fields] OR "muscle radiodensity"[All Fields] OR "dynapenia"[All Fields] OR "myopenia"[All Fields])**

Scopus

(TITLE-ABS-KEY (("sarcopenia" OR "sarcopenic" or "muscle index" OR "muscle mass" OR "muscle depletion" OR "muscular atrophy" or "muscle strength" or "muscle quality" or "muscle quantity" OR "myosteatosis" OR "muscle radiodensity" OR "dynapenia" OR "myopenia" ))) and (TITLE-ABS-KEY (("head and neck" OR "larynx" OR "laryngeal" OR "oropharynx" OR "oropharyngeal" OR "hypopharynx" OR "hypopharyngeal" OR "oral" OR "tongue" OR "parotid" OR "salivary gland" OR "nasal" OR "paranasal" ) AND ("tumor" OR "malignancy" OR "cancer")))

医中誌Web

((((("筋肉減少症"/TH or "sarcopenia"/AL) and OR&#160;/AL and "sarcopenic"/AL or "muscle index"/AL or ("筋量"/TH or "muscle mass"/AL) or "muscle depletion"/AL or ("筋萎縮症"/TH or "muscular atrophy"/AL) or ("筋力"/TH or "muscle strength"/AL) or "muscle quality"/AL or "muscle quantity"/AL and &#160;/AL or (筋肉減少症/TH or サルコペニア/AL) or (筋力/TH or 筋力/AL) or 筋指数/AL or (筋量/TH or 筋量/AL) or (筋萎縮症/TH or 筋萎縮/AL))) and ((頭頸部腫瘍/TH or 頭頸部癌/AL) or (咽頭腫瘍/TH or 咽頭癌/AL) or (喉頭腫瘍/TH or 喉頭癌/AL) or 鼻腔癌/AL or (副鼻腔腫瘍/TH or 副鼻腔癌/AL) or (上顎洞腫瘍/TH or 上顎洞癌/AL) or (唾液腺腫瘍/TH or 唾液腺癌/AL) or (耳下腺腫瘍/TH or 耳下腺癌/AL) or (顎下腺腫瘍/TH or 顎下腺癌/AL)))) and (PT=会議録除く)
